# Three-dimensional choroidal vascularity index in central serous chorioretinopathy using ultra-widefield swept-source optical coherence tomography angiography

**DOI:** 10.3389/fmed.2022.967369

**Published:** 2022-09-07

**Authors:** Qiaozhu Zeng, Lan Luo, Yuou Yao, Shu Tu, Zhi Yang, Mingwei Zhao

**Affiliations:** ^1^Department of Ophthalmology, Eye Diseases and Optometry Institute, Peking University People’s Hospital, Beijing, China; ^2^Beijing Key Laboratory of Diagnosis and Therapy of Retinal and Choroid Diseases, College of Optometry, Peking University Health Science Center, Beijing, China; ^3^TowardPi (Beijing) Medical Technology, Beijing, China

**Keywords:** central serous chorioretinopathy, three-dimensional, choroidal vascularity index, ultra-widefield swept source optical coherence tomography, vortex vein

## Abstract

**Background:**

To map and compare the three-dimensional choroidal vascularity index (3D-CVI) in eyes with unilateral central serous chorioretinopathy (CSC), fellow eyes and control eyes using ultra-widefield swept source optical coherence tomography (UWF SS-OCTA).

**Methods:**

In this prospective observational study, the 3D-CVIs were measured in 9 subfields or 1 × 1 mm grids by the UWF SS-OCTA with a viewing angle of horizontal 24 × vertical 20 mm. The proportions of vortex vein anastomoses and their corresponding CVI in the central regions were compared among the CSC, fellow and control eyes. Correlations of CVI and vascular density of the large choroidal vessel layer/choriocapillaris layer/choroidal thickness (CT) were also assessed.

**Results:**

Thirty-two eyes in 32 patients with unilateral CSC and 32 normal eyes were included in the study. The mean CVI in the eyes with CSC was significantly greater than that in the fellow eyes of CSC and control eyes (41.99 ± 3.56% vs. 40.38 ± 3.855%, *P* = 0.003; 41.99 ± 3.56% vs. 38.93 ± 4.067%, *P* = 0.004, respectively). The CVIs in superotemporal, inferotemporal and inferonasal regions were significantly higher in CSC eyes than control eyes (*P* = 0.03, *P* = 0.02, *P* = 0.008). In CSC eyes, there was a linear positive correlation between 3D-CVI and vascular density of the large choroidal vessel layer and CT in all subfields. The proportion of vortex vein anastomoses in CSC was 25/32 (78.1%), and significantly higher in fellow and control eyes (*P* < 0.001). The average central CVI was significantly higher in CSC eyes with anastomoses than in CSC eyes without anastomoses (42.8 ± 5.1% vs. 38.4 ± 2.7%, *P* = 0.039). CVIs in superior, central, inferior, superonasal, nasal and inferonasal regions were significantly correlated with vortex vein anastomoses (*P* < 0.05), regardless of CSC, fellow or healthy eyes. In addition, whether there were vortex vein anastomoses, CVI in superotemporal region was significantly higher in eyes with CSC (*P* = 0.002) and fellow eyes (*P* = 0.014), compared to control eyes. No significant correlation was found between hypertension and CVIs in the three groups.

**Conclusion:**

Remodeling of choroidal drainage routes by venous anastomosis between superior and inferior vortex veins may be common in CSC. The 3D-CVI could be a comprehensive parameter to evaluate the choroid vasculature and help understand the pathogenesis of pachychoroid spectrum disease.

## Introduction

Central serous chorioretinopathy (CSC) is a representative pachychoroid spectrum disease characterized by serous retinal detachment with or without retinal pigment epithelium (RPE) detachment (1). Abnormality in choroidal vasculature has been an established factor in the pathogenesis of CSC, manifested as delayed choroidal filling, dilated veins, intervortex venous anastomoses, and choroidal vascular hyperpermeability, leakage into interstitial or stromal space (2–5). Quantitative measurement of subretinal choroidal fluid, hyperreflective dots, and choroidal thickness has been widely investigated to be the imaging biomarkers on optical coherence tomography (OCT) in CSC (6). However, the aforementioned parameters are known to be affected by many factors (7–11). Recently, choroidal vascular index (CVI), referred to the ratio of the luminal area to the cross-sectional choroidal area, has been extensively reported to be a quantifiable and reliable choroidal parameter in CSC (12–15). Nevertheless, the two-dimensional CVI on a single B-scan is not representative of the whole choroidal vasculature.

Compared with enhanced depth imaging (EDI) spectrum-domain OCT, ultra-widefield swept source optical coherence tomography (UWF SS-OCTA) enables clinicians to visualize the full-thickness choroid in more peripheral areas of the fundus and more accurately quantify the choroidal structure (16, 17). An UWF SS-OCTA device is available from TowardPi Medical Technology (TowardPi Medical Technology Co., Ltd., Beijing, China): BM400K BMizar. With the combination of long wavelength (1,060 nm) full range swept source and 400 kHz A-scan rate, the device has capability to acquire as deep as 6 mm scan depth, scan range of horizontal 24 × vertical 20 mm and the largest field of view of 81°× 68°. Three-dimensional CVI (3D-CVI) maps could provide additional information on the choroid supply at posterior pole and periphery areas in CSC, and therefore help us better understand the pathophysiology of the disease.

Processes of vascular remodeling including vortex vein congestion and formation of inter-vortex venous anastomoses have also been recently demonstrated in eyes with pachychoroid disease, which could play an important role in CSC (18). In order to better understand the underlying mechanism of CSC, with the 3D-CVI as a metric using UWF SS-OCTA, we aim to compare the 3D-CVI in CSC, fellow and control eyes, and investigated the correlations of CVI with other choroidal parameters.

## Materials and methods

### Participants

This prospective observational study was approved by the Institutional Review Board Committee of Peking University People’s Hospital (2022PHB164-001) and adhered to the tenets of the Declaration of Helsinki. Written informed consent was obtained from all the participants.

We consecutively enrolled 32 patients who were diagnosed with treatment-naive unilateral CSC in the Department of Ophthalmology, Peking University People’s Hospital, from August 2021 to December 2021; their corresponding healthy eyes were also studied. The symptom duration after onset was within 12 months. Age-matched participants with normal eyes were included as controls.

The diagnosis of CSC was based on the presence of subretinal fluid (SRF) and/or pigment epithelium detachment (PED) on SS-OCT, with dye leakage from the RPE on FA and focal choroidal vascular hyperpermeability on the late-phase indocyanine green angiography (ICGA). Inclusion criteria of CSC were as follows: (1) Presence of SRF involving the fovea with or without PED on OCT, (2) evidence of active leakage on FA, (3) abnormal dilated choroidal vasculature on ICGA, (4) No previous treatments for CSC (including eplerenone, focal laser, intravitreal injection of anti-vascular endothelial growth factor agents, photodynamic therapy or history of surgery, etc.). Participants were excluded if: (1) Any other ocular diseases associated with SRF, such as choroidal neovascularization (CNV), polypoidal choroidal vasculopathy (PCV), diabetic retinopathy (DR), retinal vein occlusion (RVO), Coats’ disease and etc., (2) myopia with the spherical equivalent < –6 diopters, hyperopia > + 3 diopters (spherical equivalent was defined as the sum of the spherical power and half of the cylinder power), (3) any disease that may affect the quality of imaging (quality of OCT or OCTA < 7) such as cataract, high myopia, or nystagmus, (4) severe kidney or liver dysfunction and/or unstable cardiac disease, (5) pregnancy, (6) any conditions rendering patients intolerable to image acquisitions.

Details including age, gender, refraction error, best-corrected visual acuity (BCVA), axial length (AL), intraocular pressure (IOP), history of diabetes mellitus, and hypertension were recorded at the initial visit.

### Ultra-widefield swept source optical coherence tomography analysis

Participants were imaged with the 400 kHz SS-OCTA instrument (BM400K, TowardPi Medical Technology Co., Ltd., Beijing, China). Both OCT and OCTA data, were obtained with a raster scan protocol of 1,536 (horizontal) × 1,280 (vertical) B-scans, which covered an area of 24 × 20 mm centered on the fovea. The 3D-CVI was defined as the ratio of the choroidal vascular luminal volume to the total choroidal volume, which reflects the volumetric choroidal vascular density (12, 13). Choroidal thickness (CT) was set as the vertical distance from the Bruch’s membrane (BM) to the choroid-scleral interface (CSI). The density of the vessel layer was automatically calculated as the ratio of the pixel areas of the vessels divided by the total area of the regions. Large-vessel choroidal layer was the slab between the CSI and 29 μm beneath the Bruch’s membrane. Both Bruch’s membrane and CSI were identified automatically with the built-in software. We manually verified the accuracy of automatic segmentation with B-scans if necessary. When measuring the choroidal vasculature, we unified the measurement range among participants by correcting the AL-related magnification using the modified Littmann formula (Bennett procedure) (19, 20).

The measurement position was always centered at the fovea without any rotation and the data from the left eyes was horizontally flipped for statistical analysis.

**FIGURE 1:**
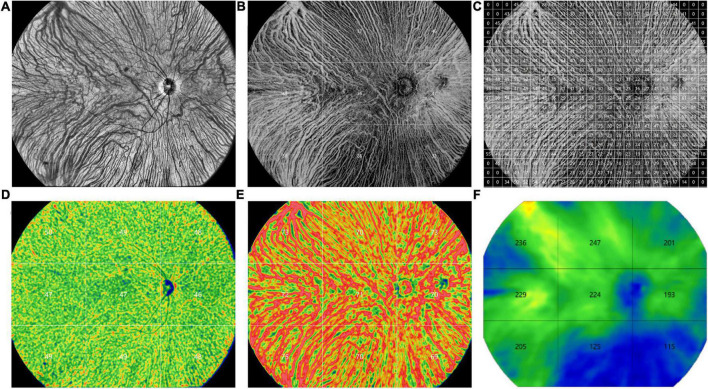
Representative ultra-widefield swept-source optical coherence tomography (UWF SS-OCTA) images obtained from a control eye. **(A)** An en face image. **(B)** The choroidal vascularity index (CVI) values in 9 subfields (superotemporal, temporal, inferotemporal, superior, central, inferior, superonasal, nasal, inferonasal). **(C)** The CVI values in grids of 1 × 1 mm. **(D)** The vascular density of the choriocapillaris layer in 9 subfields. **(E)** The vascular density of the large choroidal vessel layer in 9 subfields. **(F)** The choroidal thickness values in 9 subfields.

Similar to the method of obtaining SS-OCT images described and validated previously by Agrawal et al. (12) and Chen et al. (21), all the 3D-CVI, vascular density of the large choroidal vessel layer and choriocapillaris layer, and CT were measured and computed automatically with the built-in software for each subfield (superotemporal, temporal, inferotemporal, superior, central, inferior, superonasal, nasal, inferonasal and 1 × 1 mm grid) (Figure 1). The CVI values were also presented for grids of 1 × 1 mm to show the choroid layer as detail as possible (Figure 1C).

The presence of anastomosis between superior and inferior vortex veins was demonstrated using en face OCT and ICGA. The presence of dilated vortex veins, choroidal vascular hyperpermeability, and anatomical and functional anastomoses between the superior and inferior vortex veins were judged and confirmed by the two experienced retinal experts (YYO and ZMW). On en face large choroidal vessel layer of OCT images, anastomoses in CSC were always presented as asymmetric dilated vortex veins in the posterior pole, lost horizontal watershed between the superior and inferior vortex veins (22, 23). The proportions of vortex vein anastomoses were compared among the CSC, fellow and control eyes, as well as their corresponding CVI in the central regions.

### Statistical analysis

Statistical analysis was performed using SPSS software (version 27.0; IBM, Chicago, United States). For patient characteristics, descriptive methods, with standard summary statistics including the mean (S.D., standard deviation), median, interquartile range (IQR), and proportions were applied. Comparisons between healthy eyes and eyes with CSC were performed using the independent *t*-test for parameters with normal distribution and the Mann-Whitney *U*-test for parameters with non-normal distribution. Comparisons between eyes with CSC and fellow eyes were performed using the paired *t*-test for parameters with normal distribution and the Wilcoxon signed rank test for parameters with non-normal distribution. Pearson’ chi-square test was performed for group differences in dichotomous variables. Correlation between the CVI, blood flow density of large choroid vessels and CT were performed using Pearson correlation test or Spearman correlation test. Multiple linear regression analysis was applied to calibrate the influence of important factors on CVI when comparing the CVIs among eyes with CSC, fellow eyes and healthy eyes. The coefficient of variation (CV) was calculated as the standard deviation divided by the mean value. *P*-values of < 0.05 were considered statistically significant.

## Results

In our study, 32 diseased eyes and 32 fellow eyes of 32 patients (26 males, 6 females) with unilateral CSC and 32 eyes of 32 normal subjects (23 males, 9 females) were examined. The mean symptom duration of CSC patients was 3.1 ± 2.9 months. No significant difference in gender distribution, age, spherical equivalent, and AL, IOP was found among the CSC eyes, fellow eyes, and control eyes (Tables 1, 2).

**TABLE 1:** Characteristics of patients with CSC and healthy subjects.

Clinical parameters	Patients with CSC	Healthy subjects	*P-value*
No. (male/female)	32 (26/6)	32 (23/9)	0.376^ǁ^
No. of eyes	32	32	NA
Age, years, mean ± SD	45.03 ± 11.394	46.79 ± 8.510	0.484^ǁǁ^
Hypertension, *n* (%)	5 (15.6)	1 (3.1)	0.09^ǁ^
Diabetes, *n* (%)	1 (3.1)	3 (9.4)	0.302^ǁ^

CSC, central serous chorioretinopathy; NA, not applicable; SD, standard deviation. ^ǁ^P-value determined by chi-square test. ^ǁǁ^P-value determined by independent-samples t-test.

**TABLE 2:** Comparisons of parameters among CSC eyes, fellow eyes and healthy eyes.

	CSC eyes	Fellow eyes	Healthy eyes	*P-value* ^a^	*P-value* ^b^	*P-value* ^c^
Best corrected visual acuity in logMAR	0.2 ± 0.3	0.1 ± 0.2	0 ± 0.0	0.068^#^	<0.001^‡^*	<0.001^‡^*
Intraocular pressure, mmHg	14.3 ± 2.7	14.1 ± 2.5	15.6 ± 3.8	0.506^#^	0.146^ǁ^	0.203^ǁ^
Axial length, mm	23.8 ± 1.0	23.9 ± 1.1	24.1 ± 1.2	0.162^#^	0.690^ǁ^	0.469^ǁ^
Spherical equivalent, diopters	–0.65 ± 2.0	–1.0 ± 2.4	–1.15 ± 1.9	0.871^#^	0.672^ǁ^	0.374^‡^

CSC, central serous chorioretinopathy; logMAR, Logarithm of the Minimum Angle of Resolution; SD, standard deviation. Data are presented as mean ± standard deviations. *Statistically significant. ^*a*^Comparisons of ocular factors between diseased and fellow eyes of patients with CSC. ^*b*^Comparisons of ocular factors between healthy eyes and fellow eyes of patients with CSC. ^*c*^Comparisons of ocular factors between healthy eyes and diseased eyes of patients with CSC. ^ǁ^P-value determined by independent-samples t-test. ^‡^P-value determined by Mann-Whitney U-test. ^#^P-value determined by Wilcoxon signed-rank test.

### Comparison of choroidal vascularity index

**FIGURE 2:**
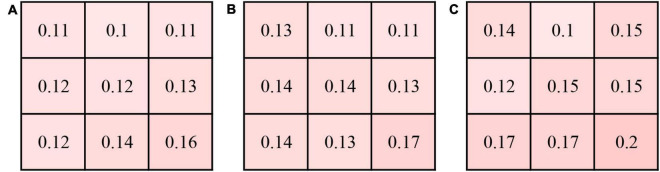
Coefficient of variation (CV) of the CVI in 9 subfields in the eyes with central serous chorioretinopathy (CSC) **(A)**, fellow eyes **(B)** and controls eyes **(C)**.

Subfield CVIs in the eyes with CSC, fellow eyes, and control eyes (superotemporal, temporal, inferotemporal, superior, central, inferior, superonasal, nasal, inferonasal) were summarized in Table 3. The mean CVI in the eyes with CSC was significantly greater than that in the fellow eyes (41.99 ± 3.56% vs. 40.38 ± 3.855%, *P* = 0.003) and control eyes (41.99 ± 3.56% vs. 38.93 ± 4.067%, *P* = 0.004). In superotemporal (*P* = 0.03), superior (*P* = 0.024), central (*P* = 0.004), inferior (*P* = 0.027), superonasal (*P* = 0.02) and inferonasal (*P* = 0.008) regions, the CVI of the eyes with CSC were statistically higher than that of control eyes. Compared with the CVI values of the fellow eyes, those of eyes with CSC were significantly greater in temporal (*P* = 0.008), superior (*P* = 0.02), central (*P* = 0.001) and inferior (*P* = 0.009) regions. Subfield CVIs, with the exception of the superotemporal subfield, were insignificantly different between the controls and the fellow eyes. The CV of the CVI in the controls eyes was 0.175, and the CV of subfield CVIs in eyes with CSC, the fellow eyes and control eyes was shown in Figure 2.

**TABLE 3:** Comparisons of CVI between healthy subjects and patients with CSC.

		Patients with CSC				
CVI%	Healthy subjects	Fellow eyes	Diseased eyes	*P-value* ^a^	*P-value* ^b^	*P-value* ^c^
Mean	38.93 ± 4.067	40.38 ± 3.855	41.99 ± 3.56	0.118^‡^	0.004^‡^	0.003^#^
**Subfields**						
Superotemporal	41.22 ± 5.813	44.03 ± 5.631	45.38 ± 4.989	0.025^‡^	0.003^ǁ^	0.161^¶^
Temporal	42.03 ± 5.102	41.28 ± 5.958	43.19 ± 5.165	0.591^‡^	0.371^ǁ^	0.008^¶^
Inferotemporal	42.59 ± 7.233	43.91 ± 6.342	45.50 ± 5.322	0.443^ǁ^	0.148^‡^	0.081^¶^
Superior	36.38 ± 3.731	37.94 ± 4.103	39.09 ± 3.675	0.116^ǁ^	0.024^‡^	0.020^¶^
Central	37.91 ± 5.613	39.47 ± 5.483	41.81 ± 4.954	0.264^ǁ^	0.004^‡^	0.001^¶^
Inferior	33.69 ± 5.828	35.06 ± 4.662	36.75 ± 4.964	0.301^ǁ^	0.027^ǁ^	0.009^¶^
Superonasal	41.75 ± 6.101	43.69 ± 4.980	45.06 ± 4.931	0.169^ǁ^	0.020^ǁ^	0.114^¶^
Nasal	41.97 ± 6.301	42.97 ± 5.373	44.06 ± 5.691	0.686^ǁ^	0.226^ǁ^	0.319^#^
Inferonasal	32.84 ± 6.481	35.03 ± 5.872	37.09 ± 5.827	0.162^ǁ^	0.008^ǁ^	0.113^#^

CSC, central serous chorioretinopathy. ^*a*^Comparisons between healthy eyes and fellow eyes of patients with CSC. ^*b*^Comparisons between healthy eyes and eyes of patients with CSC. ^*c*^Comparisons between fellow and diseased eyes of patients with CSC. ^#^P-value determined by Wilcoxon signed-rank test. ^¶^P-value determined by paired t-test. ^‡^P-value determined by Mann-Whitney U-test. ^ǁ^P-value determined by independent-samples t-test.

Depiction of the detailed CVI values of each 1 × 1 mm grid was represented in Figure 1C. In each group, the CVI values were higher in the nasal and temporal regions than the superior and inferior regions. The CVI in the eyes with CSC was significantly greater than that in the controls in several grids, mainly in the posterior pole (*P* < 0.05) (Supplementary Figure 1). The CV of the CVI in the controls eyes was 0.27 (detailed information was shown in Supplementary Figure 2).

### Correlations of choroidal vascularity index and vascular density of the large choroidal vessel layer/choriocapillaris layer/choroidal thickness

**FIGURE 3:**
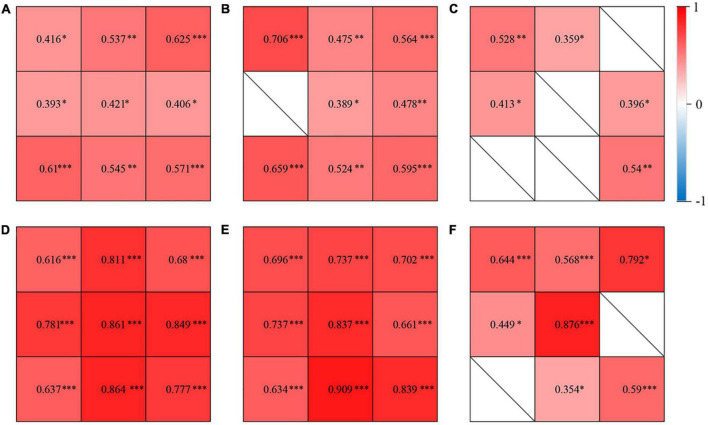
Correlations of CVI and vascular density of the large choroidal vessel layer in the eyes with central serous chorioretinopathy (CSC) **(A)**, the fellow eyes of CSC **(B)**, and control eyes **(C)**. Correlations of CVI and choroidal thickness (CT) in the eyes with CSC **(D)**, the fellow eyes of CSC **(E)**, and control eyes **(F)**. The correlation coefficient was represented as the value in 9 subfields. ^∗^*P*< 0.05; ^∗∗^*P <* 0.01; ^∗∗∗^*P* < 0.001.

**FIGURE 4:**
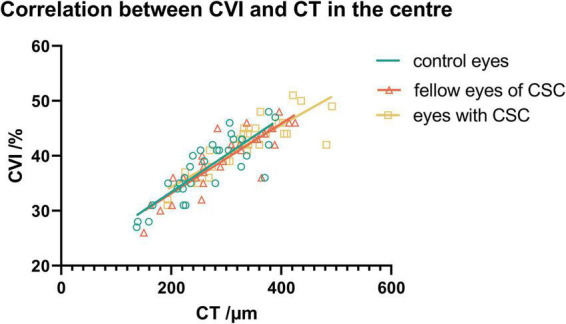
The association between CVI and CT in the central subfield in the three groups.

In the CSC, fellow and control groups, CVI showed no significant correlations with the vascular density of choriocapillaris in any subfields (*P* > 0.05); while CVI showed a significant positive association with the vascular density of the large choroidal vessel layer in several subfields (Figures 3A–C). Also, CVI showed a significant association with CT in several subfields (Figures 3D–F). In particular, in the eyes with CSC, CVI had a high correlation with CT in superior (*r* = 0.811, *P* < 0.001), central (*r* = 0.861, *P* < 0.001), inferior (*r* = 0.864, *P* < 0.001) and nasal (*r* = 0.849, *P* < 0.001) regions (Figure 3D); in fellow eyes, CVI had a high correlation with CT in central (*r* = 0.837, *P* < 0.001), inferior (*r* = 0.909, *P* < 0.001) and inferonasal (*r* = 0.839, *P* < 0.001) regions (Figure 3E); and in control eyes, it was in central (*r* = 0.876, *P* < 0.001) region (Figure 3F) that CVI had a high correlation with CT. No matter in which group, CVI in the central showed a high association with CT in the central (Figure 4). Detailed information about the vascular density of the large choroidal vessel layer and choriocapillaris layer, and CT was shown in Supplementary Table 1.

### Vortex vein anastomoses and choroidal vascularity index

**FIGURE 5:**
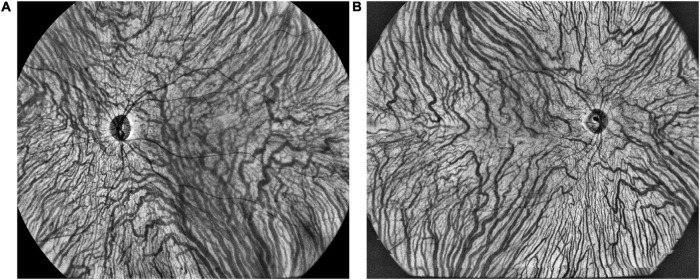
The representative vortex vein anastomoses in a CSC eye **(A)** and symmetric vortex vein in a healthy eye **(B)** on en face large choroidal vessel layer.

The representative vortex vein anastomose in a CSC eye and its control in a healthy eye were presented in Figure 5. There were 25 (78.1%), 15 (46.9%), and 5 (15.3%) eyes with vortex vein anastomoses in CSC, fellow and control eyes (*P* < 0.001). As shown in Table 4, in all the three groups, the average central CVI in eyes with anastomoses was significantly higher than those without anastomoses (in the group of CSC eyes: 42.8 ± 5.1% vs. 38.4 ± 2.7%, *P* = 0.039; in the group of fellow eyes: 43.3 ± 2.8% vs. 36.1 ± 5.1%, *P* < 0.001; in the control group: 42.8 ± 3.1% vs. 37.0 ± 5.5%, *P* = 0.031; respectively).

**TABLE 4:** Central CVI in eyes with or without anastomoses in CSC, fellow and control groups.

Groups	CVI in eyes with anastomoses, mean ± SD, %	CVI in eyes without anastomoses, mean ± SD, %	*P-value*
CSC	42.8 ± 5.1	38.4 ± 2.7	0.039
Fellow	43.3 ± 2.8	36.1 ± 5.1	<0.001
Control	42.8 ± 3.1	37.0 ± 5.5	0.031

Results of multiple regressions were summarized in Table 5. It was revealed that CVIs in superior, central, inferior, superonasal, nasal and inferonasal regions were significantly correlated with vortex vein anastomoses (*P* < 0.05), regardless of CSC, fellow or healthy eyes. In addition, whether there were vortex vein anastomoses, CVI in superotemporal region was significantly higher in eyes with CSC (*P* = 0.002) and fellow eyes (*P* = 0.014), compared to control eyes. In addition, no significant correlation was found between hypertension and CVIs in the three groups.

**TABLE 5:** Results of multiple regression analysis of association between CVI and other parameters.

		Standardized β coefficient (95% CI)	*P-value*
**Mean CVI**			
Group	Fellow eyes	0.071 (–1.312, 2.514)	0.534
	Diseased eyes	0.137 (–1.027, 3.344)	0.295
Hypertension		–0.077 (–3.348, 1.416)	0.423
Vortex vein anastomoses		0.388 (1.317, 4.854)	< 0.001^#^
**Superotemporal**			
Group	Fellow eyes	0.302 (0.745, 6.525)	0.014^#^
	Diseased eyes	0.452 (2.134, 8.736)	0.002^#^
Hypertension		–0.183 (–6.857, 0.340)	0.075
Vortex vein anastomoses		–0.117 (–4.000, 1.344)	0.326
**Temporal**			
Group	Fellow eyes	–0.108 (–4.054, 1.575)	0.384
	Diseased eyes	–0.016 (–3.395, 3.035)	0.912
Hypertension		–0.132 (–5.744, 1.265)	0.208
Vortex vein anastomoses		0.228 (–0.140, 5.064)	0.063
**Inferotemporal**			
Group	Fellow eyes	0.067 (–2.490, 4.290)	0.599
	Diseased eyes	0.148 (–1.875, 5.870)	0.308
Hypertension		–0.016 (–4.532, 3.910)	0.884
Vortex vein anastomoses		0.113 (–1.690, 4.578)	0.362
**Superior**			
Group	Fellow eyes	0.127 (–0.872, 2.996)	0.278
	Diseased eyes	0.166 (–0.824, 3.594)	0.216
Hypertension		–0.166 (–4.462, 0.354)	0.094
Vortex vein anastomoses		0.307 (0.636, 4.212)	0.008^#^
**Central**			
Group	Fellow eyes	–0.004 (–2.564, 2.477)	0.973
	Diseased eyes	0.029 (–2.545, 3.213)	0.818
Hypertension		–0.084 (–4.590, 1.686)	0.361
Vortex vein anastomoses		0.518 (3.390, 8.050)	< 0.001^#^
**Inferior**			
Group	Fellow eyes	0.018 (–2.436, 2.837)	0.880
	Diseased eyes	0.052 (–2.430, 3.593)	0.702
Hypertension		–0.007 (–3.390, 3.175)	0.948
Vortex vein anastomoses		0.362 (1.363, 6.238)	0.003^#^
**Superonasal**			
Group	Fellow eyes	0.037 (–2.226, 3.091)	0.747
	Diseased eyes	0.013 (–2.886, 3.187)	0.922
Hypertension		–0.001 (–3.325, 3.295)	0.993
Vortex vein anastomoses		0.442 (2.364, 7.280)	< 0.001^#^
**Nasal**			
Group	Fellow eyes	–0.054 (–3.556, 2.223)	0.648
	Diseased eyes	–0.109 (–4.633, 1.968)	0.425
Hypertension		0.029 (–3.067, 4.129)	0.770
Vortex vein anastomoses		0.443 (2.448, 7.792)	<0.001^#^
**Inferonasal**			
Group	Fellow eyes	0.085 (–2.042, 4.297)	0.880
	Diseased eyes	0.156 (–1.567, 5.674)	0.702
Hypertension		0.011 (–3.733, 4.160)	0.915
Vortex vein anastomoses		0.266 (0.377, 6.237)	0.027^#^

Significative P-values are marked with ^#^.

## Discussion

Our study quantitatively analyzed the three-dimensional alterations of choroidal vascularity using a horizontal 24 × vertical 20 mm UWF SS-OCTA scan. Significant differences were found in 3D-CVI among eyes with CSC, fellow and control eyes. In addition, we observed widespread vortex vein anastomoses in eyes with CSC, in which high 3D-CVI was demonstrated in the macular region. In CSC and fellow eyes, there was a linear positive correlation between 3D-CVI and vascular density of the large choroidal vessel layer and CT.

CVI is a widely focused and reliable metric for the quantification of both the vascular and interstitial components of the choroid. It has little variability and is not affected by physiological factors, such as AL, IOP, age, and systolic blood pressure (24). Similar to the published study (24), no significant correlation was found between hypertension and CVIs in the three groups. The CVI was not affected by the systemic factors, which further supported the reliability of CVI for the assessment of choroid. Measuring CVI over a large area will obviously be closer to the true measurement (24). Our study may evaluate the choroid vasculature more accurately compared with previous studies concentrating on the macular area with two-dimensional images.

Two-dimensional CVI has been investigated to be 64–67% in normal individuals and alter in CSC from 53 to 70% due to the heterogeneity of CVI definition, technologies and designs in various previous studies (12, 24–29). Yang et al. had reported the mean 3D-CVI in the 12 × 12 mm scan area was 0.35 in the eyes with CSC and 0.30 in the control eyes (13). In our study, the mean CVI in CSC eyes was 42%, statistically higher than that in control eyes (39%), consistent with previous studies (12, 13, 27). Similar to the study by Yang et al. (13), the CVIs in drainage route of choroidal veins, including superotemporal, inferotemporal and inferonasal were significantly higher in CSC eyes, indicating the congestion of vortex vein may play a role in the pathogenesis. Interestingly, the vortex vein anastomoses were more commonly found in CSC than in controls. The central CVIs in eyes with vortex vein anastomoses were also higher than eyes without anastomoses. In addition, we calibrated the influence of important parameters on CVI for the first time. Vortex vein anastomoses were the main factor for increased CVIs in all eyes and the deep-seated factor for higher CVI in CSC eyes, which further supported the reason for more anastomoses in CSC eyes than healthy controls. The choroidal congestion was ameliorated by the new drainage route established by the anastomosis developing between the superior and inferior vortex veins (22). On the other hand, we found that regardless of vortex vein anastomoses, the CVI in superotemporal region was statistically higher in eyes with CSC and fellow eyes, compared to control eyes, which confirmed the congestion status of dominant vortex veins in CSC eyes. Therefore, we speculated that in CSC eyes, the asymmetric dominant vortex veins may be an intrinsic predisposing factor for congestion in CSC, and then to ameliorate the congestion, the anastomosis between the superior and inferior vortex veins more frequently developed than in fellow and healthy eyes, manifested as higher CVIs in CSC. Pang et al. proposed that dilatation of the ampulla in the dominant vortex vein suggests outflow disturbance through the scleral tunnel, which might be narrowed by the thickened sclera (30). We will carry out further research in the future to testify the causal relationships among sclera thickness, asymmetric dominant vortex veins, anastomosis, and CVIs. In all, our findings may further support the recent understanding of the pathogenesis of typical CSC, supposedly involving congestion due to the impaired drainage of the affected vortex veins (22, 23, 31–34). We speculate that vortex vein congestion may lead to anastomosis between the superior and inferior vortex veins. Remodeling of choroidal drainage routes by venous anastomosis between superior and inferior vortex veins may be common in CSC (23).

In this study, we found that CVI showed a significant positive correlation with CT in eyes with CSC, and the correlation coefficient was relatively high in several subfields. CVI had a high correlation with CT in the eyes with CSC in superior, central, inferior and nasal regions, in fellow eyes in central, inferior and inferonasal regions, and in control eyes in central region. Consistent with previous research, in Ishikura et al.’s study (34), compared with that in control eyes, the CT in eyes of patients with CSC were significantly greater in all subfields. They proposed that in areas with dilated vortex veins, choroidal thickening was observed from the vicinity of the vortex vein ampulla to the macula along the course of the veins. Yang et al. reported that the eyes with CSC and the fellow eyes had significantly higher CVI values at the posterior pole and the drainage routes of choroidal veins, including central, superior and inferior regions (13). In Singh et al.’s study, CVI and CT varied significantly in different segments in horizontal and vertical meridian in CSCR and fellow eyes (35). We speculated that in the drainage routes of choroidal veins, it is mainly vascular component that increases and leads to the thicker choroid and higher CVI, which further confirm remodeling of choroidal drainage routes in superior and inferior vortex veins and venous anastomosis in the macular region. From this perspective, the local factors of the affected vortex vein may be involved in the pathogenesis of CSC.

Interestingly, the subfield CVI in fellow eyes was higher than that in control eyes mostly, although the difference in CVI was not seen to be significantly different. Previous studies shown that fellow eyes and eyes with CSC had similar changes in several parameters, including the CT, blood flow, and CVI, suggesting that alterations in choroidal vasculature may essentially be bilateral or systemic (12, 36, 37). We also observed sizeable vortex vein anastomoses in fellow eyes, significantly higher than in control eyes. These changes suggested there be systemic risk factors making these patients prone to having CSC.

Singh et al. (35) found that the CVI was variable in various quadrants using several widefield single B-scans. Therefore, repeatability of CVI needs to be validated in a certain situation. The coefficient of variation of mean 3D-CVI in our study for the control eyes was 0.175, which was similar to previous study (13), suggesting that 3D-CVI might be a metric with good repeatability.

Similar to Singh et al.’s study (26), we found CSC eyes and fellow eyes showed worse best-corrected visual acuity (BCVA) than the healthy control eyes. There could be several explanations. In the unaffected eyes of CSC patients, non-specific RPE changes have been reported to be pachychoroid pigment epitheliopathy (PPE), as a forme fruste of CSC (38, 39). In addition, some of the unaffected eyes in CSC can be uncomplicated pachychoroid (UCP) with choroidal thickness of more than 300 μm (40). Therefore, the fellow eyes of CSC patients may serve as PPE, UCP or healthy eyes (41). In our study, not all the fellow eyes were completely normal, including 54.3% of fellow eyes with PPE, 26.1% with UCP. The BCVA of some fellow eyes may be worse than that of the healthy eyes. We will carry out further research about clinical and choroidal characteristics of different groups (PPE, UCP and normal) of fellow eyes in CSC patients in the future. On the other hand, the heterogeneity of included patients and controls in different studies could not be neglected. The limited sample size should be also taken into consideration when interpretating the BCVA among the three groups (CSC, fellow and control eyes).

There were several limitations in our study. The first was the small sample size due to our strict inclusion criteria. Further studies with larger sample sizes are needed. CSC patients were not categorized due to sample size. Meanwhile, we would conduct a long-term longitudinal study in the future, which may help to understand the long-term alteration in choroidal vascularity with the progression or treatments of CSC. Third, we used SS-OCTA with a viewing angle of horizontal 24 × vertical 20 mm. However, the vortex vein ampullae could not be simultaneously captured in the viewing angle. We determined the eyes with or without vortex vein anastomoses based on previously reported methods, which may be subjective. Despite of the aforementioned drawbacks, there were also several advantages and novelties in our study. One is the prospective nature. In addition, widefield 3D-CVIs of choroid in CSC have never been reported yet to support the view that congestion of vortex vein may play a role in the pathogenesis. Moreover, we firstly evaluate the relationships among different choroid parameters using ultra-widefield SS-OCTA, and proposed that 3D-CVI was a reliable imaging marker for assessing CSC. The imaging protocol applied in this study will facilitate the elucidation of the physiology of the choroidal circulation and pathologies of CSC and other pachychoroid spectrum diseases.

## Conclusion

In conclusion, increased CVI in superotemporal, inferotemporal and inferonasal regions in eyes with CSC suggests congestion of vortex vein may play a role in the pathogenesis. Remodeling of choroidal drainage routes by venous anastomosis between superior and inferior vortex veins may be common in CSC. The 3D-CVI could be an accurate and comprehensive parameter to evaluate the choroid vasculature and help understand the pathogenesis of pachychoroid spectrum disease.

## Data availability statement

The original contributions presented in this study are included in the article/Supplementary material, further inquiries can be directed to the corresponding author.

## Ethics statement

The studies involving human participants were reviewed and approved by the Institutional Review Board Committee of Peking University People’s Hospital (2022PHB164-001). The patients/participants provided their written informed consent to participate in this study.

## Author contributions

MZ: conceptualization, funding acquisition, resources, supervision, and writing—review and editing. QZ, LL, YY, and ST: data curation. QZ: formal analysis. YY: investigation. QZ and LL: methodology and roles/writing—original draft. QZ and YY: project administration. LL: software. YY and ST: validation. QZ, LL, and ZY: visualization. All authors contributed to the article and approved the submitted version.
