# Supplementary material for: Three-dimensional choroidal vascularity index in central serous chorioretinopathy using ultra-widefield swept-source optical coherence tomography angiography
Source: Front Med (Lausanne). 2022 Sep 7;9:967369. doi: 10.3389/fmed.2022.967369 (PMC9490028; doi:10.3389/fmed.2022.967369)
Supplement: Supplementary file 1 [file Data_Sheet_1.docx]

Supplementary Material

# Supplementary Figures and Tables

## Supplementary Figures

**
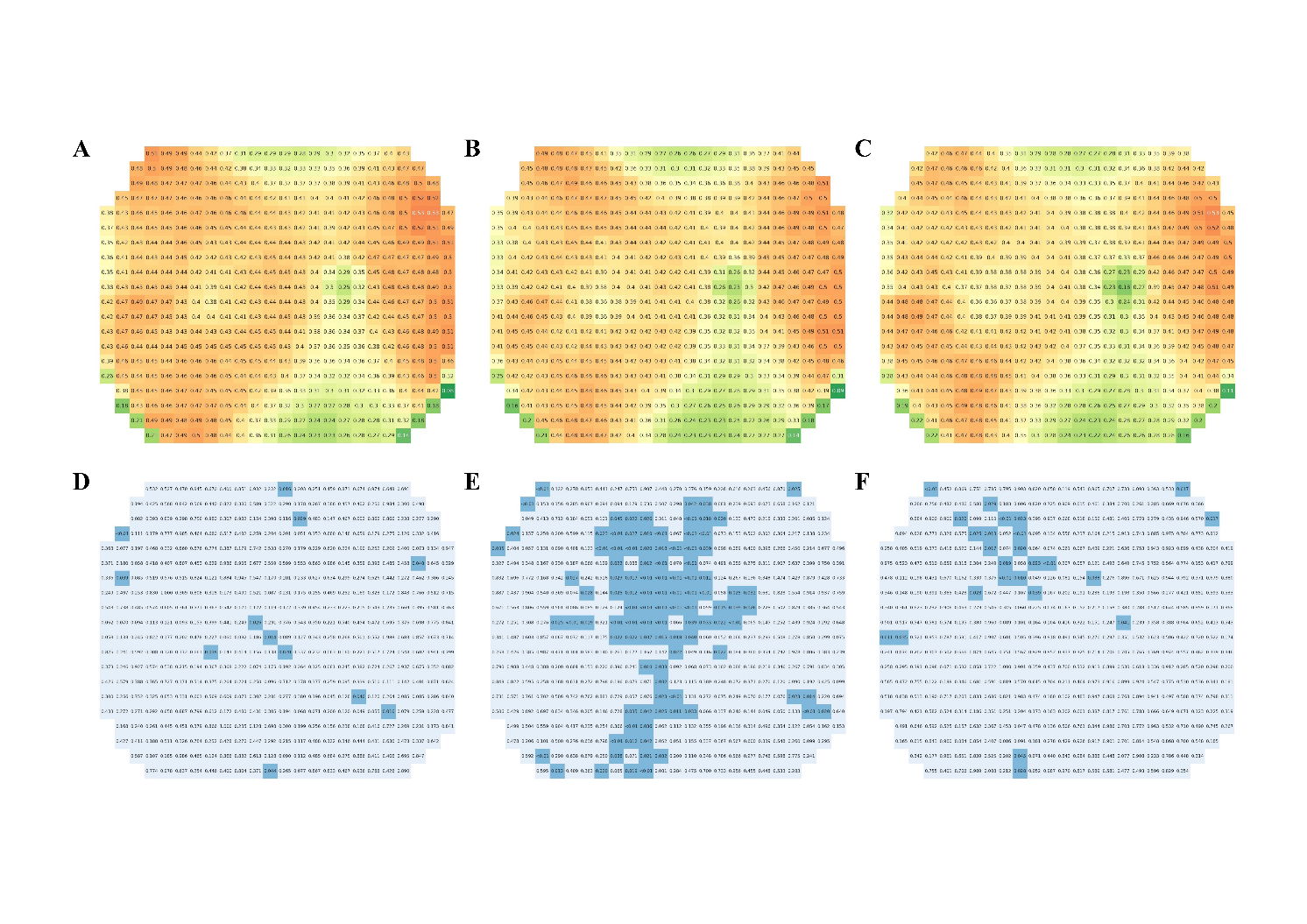
**

**Supplementary Figure 1.** Mean choroidal vascularity index (CVI) values in horizontal 24 x vertical 20 mm grids in eyes with central serous chorioretinopathy (CSC) **(A)**, fellow eyes of CSC **(B)** and control eyes**(C)**, with a different background color in each grid to distinguish various values of the CVI. The P values for the comparisons of CVI values at the same location in horizontal 24 x vertical 20 mm grids between the eyes with CSC and fellow eyes **(D)**, between the eyes with CSC and control eyes **(E)** and between the fellow eyes of CSC and control eyes **(F)**. The grids with P values less than 0.05 were indicated with a blue background.


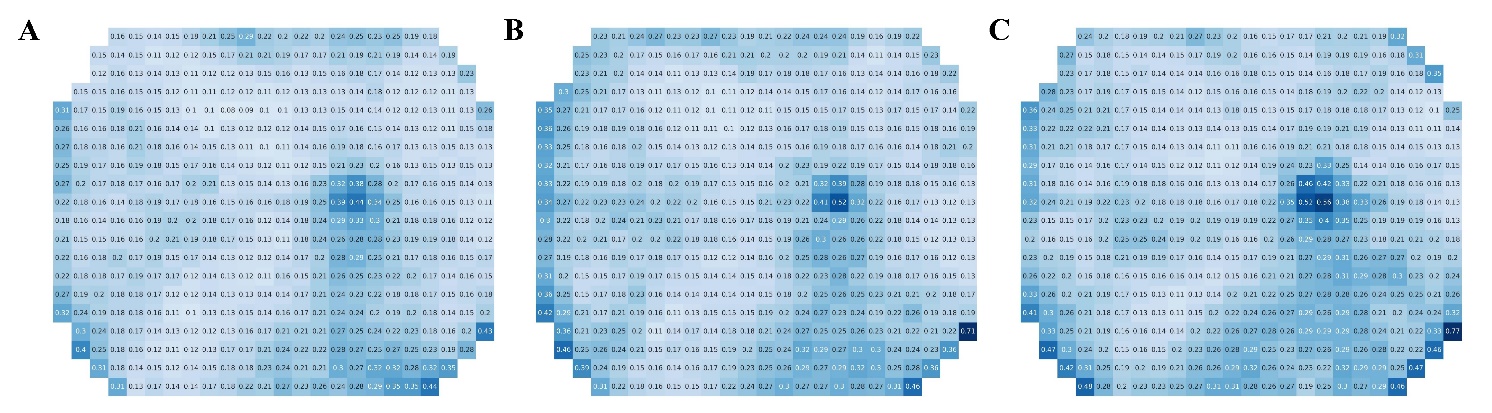


**Supplementary Figure 2.** Coefficient of variation (CV) of the CVI grids of 1×1mm in the eyes of central serous chorioretinopathy (CSC) **(A)**, fellow eyes of CSC **(B)** and controls eyes **(C)**.

## Supplementary Tables

**Supplementary Table 1.** The vascular density of the large choroidal vessel layer and choriocapillaris layer, and choroidal thickness in the eyes with CSC, fellow eyes of CSC and the control eyes

| \| Supplementary Table 1 Comparisons of choroidal vasculature in nine regions among CSC eyes, fellow eyes and healthy control eyes. \| \| \| \| \| \| \| \| \| --- \| --- \| --- \| --- \| --- \| --- \| --- \| --- \| \|  \|  \| healthy eyes \| fellow eyes \| diseased eyes \| P-value^1^ \| P-value^2^ \| P-value^3^ \| \| Large-choroidal vessel density, %, mean±SD \| \| \| \|  \|  \|  \|  \| \|  \| Mean \| 65.0±3.8 \| 66.3±4.9 \| 67.6±4.0 \| 0.002* \| 0.212 \| 0.024* \| \|  \| Superotemporal \| 64.8±5.1 \| 67.7±6.3 \| 68.9±5.9 \| 0.132 \| 0.011* \| 0.0004* \| \|  \| Temporal \| 66.6±3.4 \| 65.9±4.7 \| 67.6±3.8 \| 0.005* \| 0.730 \| 0.288 \| \|  \| Inferotemporal \| 64.3±5.6 \| 66.8±6.2 \| 68.7±4.6 \| 0.06 \| 0.103 \| 0.003* \| \|  \| Superior \| 65.4±3.8 \| 65.8±4.5 \| 66.3±3.8 \| 0.658 \| 0.231 \| 0.190* \| \|  \| Central \| 66.2±3.9 \| 66.5±5.0 \| 67.7±4.5 \| 0.009* \| 0.492 \| 0.143* \| \|  \| Inferior \| 65.1±4.6 \| 65.4±4.8 \| 66.7±4.3 \| 0.008* \| 0.475 \| 0.118 \| \|  \| Superonasal \| 64.7±5.0 \| 67.6±5.9 \| 68.2±4.8 \| 0.444 \| 0.02* \| 0.009* \| \|  \| Nasal \| 65.4±5.0 \| 66.3±4.9 \| 67.5±3.6 \| 0.014* \| 0.313 \| 0.037* \| \|  \| Inferonasal \| 61.8±5.8 \| 65.1±6.1 \| 67.2±4.8 \| 0.014* \| 0.053 \| 0.0002* \| \| Choroidal thickness, um, mean±SD \| \| \|  \|  \|  \|  \|  \| \|  \| Superotemporal \| 218.8±50.8 \| 262.8±70.2 \| 284.6±68.0 \| 0.031* \| 0.010* \| 0.0001* \| \|  \| Temporal \| 218.7±46.0 \| 244.8±63.5 \| 258.3±60.0 \| 0.003* \| 0.101 \| 0.007* \| \|  \| Inferotemporal \| 202.7±60.0 \| 228.8±67.1 \| 239.7±61.4 \| 0.041* \| 0.103 \| 0.014* \| \|  \| Superior \| 254.6±50.9 \| 282.4±69.5 \| 306.0±66.6 \| 0.042* \| 0.127 \| 0.003* \| \|  \| Central \| 266.4±70.0 \| 299.3±75.5 \| 332.8±75.9 \| 0.001* \| 0.082 \| 0.001* \| \|  \| Inferior \| 191.5±53.5 \| 221.9±65.0 \| 237.5±66.3 \| 0.142 \| 0.089 \| 0.005* \| \|  \| Superonasal \| 238.0±62.7 \| 251.9±72.9 \| 267.4±74.3 \| 0.138 \| 0.493 \| 0.126 \| \|  \| Nasal \| 218.0±59.0 \| 241.2±79.7 \| 254.0±89.6 \| 0.005* \| 0.361 \| 0.173 \| \|  \| Inferonasal \| 145.2±31.2 \| 172.3±70.3 \| 172.4±53.1 \| 0.068 \| 0.219 \| 0.044* \| \| Choriocapillaris density, %, mean±SD \| \| \|  \|  \|  \|  \|  \| \|  \| Mean \| 53.7±5.1 \| 54.3±4.7 \| 54.7±4.5 \| 0.114 \| 0.577 \| 0.397 \| \|  \| Superotemporal \| 53.7±6.8 \| 54.8±5.6 \| 55.2±6.1 \| 0.303 \| 0.531 \| 0.430 \| \|  \| Temporal \| 54.4±3.7 \| 53.3±4.4 \| 53.9±4.4 \| 0.186 \| 0.236 \| 0.615 \| \|  \| Inferotemporal \| 50.7±7.0 \| 52.3±6.0 \| 53.1±4.9 \| 0.435 \| 0.416 \| 0.242 \| \|  \| Superior \| 56.4±4.4 \| 55.8±5.0 \| 55.8±4.8 \| 0.643 \| 0.684 \| 0.517 \| \|  \| Central \| 54.6±4.2 \| 54.2±4.6 \| 54.5±4.6 \| 0.685 \| 0.823 \| 0.919 \| \|  \| Inferior \| 55.8±4.5 \| 55.2±4.8 \| 55.4±4.5 \| 0.862 \| 0.626 \| 0.587 \| \|  \| Superonasal \| 52.8±7.0 \| 55.8±6.1 \| 55.6±5.4 \| 0.872 \| 0.056 \| 0.079 \| \|  \| Nasal \| 54.4±4.0 \| 53.9±4.4 \| 53.9±4.5 \| 0.871 \| 0.861 \| 0.736 \| \|  \| Inferonasal \| 51.8±6.8 \| 53.4±5.9 \| 54.7±5.2 \| 0.107 \| 0.381 \| 0.093 \| \| Data are presented as means±standard deviations unless otherwise indicated.  CSC, central serous chorioretinopathy; SD, standard deviation  * statistically significant \| \| \| \| \| \| \|  \| \| ^1^Comparisons of ocular factors between diseased and fellow eyes of patients with CSC were performed using the paired t test for parameters with normal distribution and using the Wilcoxon matched-pairs signed rank test for parameters with nonnormal distribution. \| \| \| \| \| \| \| \| \| ^2^Comparisons of ocular factors between healthy eyes and fellow eyes of patients with CSC were performed using the independent t test for parameters with normal distribution and using the Mann Whitney U test for parameters with nonnormal distribution. \| \| \| \| \| \| \| \| \| ^3^Comparisons of ocular factors between healthy eyes and diseased eyes of patients with CSC were performed using the unpaired t test for parameters with normal distribution and using the Mann Whitney U test for parameters with nonnormal distribution. \| \| \| \| \| \| \| \| \|  \| \| \| \| \| \| \| \| |
| --- | --- | --- | --- | --- | --- | --- | --- | --- | --- | --- | --- | --- | --- | --- | --- | --- | --- | --- | --- | --- | --- | --- | --- | --- | --- | --- | --- | --- | --- | --- | --- | --- | --- | --- | --- | --- | --- | --- | --- | --- | --- | --- | --- | --- | --- | --- | --- | --- | --- | --- | --- | --- | --- | --- | --- | --- | --- | --- | --- | --- | --- | --- | --- | --- | --- | --- | --- | --- | --- | --- | --- | --- | --- | --- | --- | --- | --- | --- | --- | --- | --- | --- | --- | --- | --- | --- | --- | --- | --- | --- | --- | --- | --- | --- | --- | --- | --- | --- | --- | --- | --- | --- | --- | --- | --- | --- | --- | --- | --- | --- | --- | --- | --- | --- | --- | --- | --- | --- | --- | --- | --- | --- | --- | --- | --- | --- | --- | --- | --- | --- | --- | --- | --- | --- | --- | --- | --- | --- | --- | --- | --- | --- | --- | --- | --- | --- | --- | --- | --- | --- | --- | --- | --- | --- | --- | --- | --- | --- | --- | --- | --- | --- | --- | --- | --- | --- | --- | --- | --- | --- | --- | --- | --- | --- | --- | --- | --- | --- | --- | --- | --- | --- | --- | --- | --- | --- | --- | --- | --- | --- | --- | --- | --- | --- | --- | --- | --- | --- | --- | --- | --- | --- | --- | --- | --- | --- | --- | --- | --- | --- | --- | --- | --- | --- | --- | --- | --- | --- | --- | --- | --- | --- | --- | --- | --- | --- | --- | --- | --- | --- | --- | --- | --- | --- | --- | --- | --- | --- | --- | --- | --- | --- | --- | --- | --- | --- | --- | --- | --- | --- | --- | --- | --- | --- | --- | --- | --- | --- | --- | --- | --- | --- | --- | --- | --- | --- | --- | --- | --- | --- | --- | --- | --- | --- | --- | --- | --- | --- | --- | --- | --- | --- | --- | --- | --- | --- | --- | --- | --- | --- | --- | --- | --- | --- | --- | --- | --- | --- | --- | --- | --- | --- | --- | --- | --- | --- | --- | --- | --- | --- | --- | --- |
